
JOURNAL INFORMATION
==============================
NLM Title Abbreviation: J Can Assoc Gastroenterol
Journal ID: J Can Assoc Gastroenterol
NLM Journal ID: 101738684
Journal ID: jcag

Journal of the Canadian Association of Gastroenterology

ISSN: 2515-2084
EISSN: 2515-2092
Publisher: Oxford University Press

ARTICLE INFORMATION
==============================
PMCID: PMC8859287
DOI: 10.1093/jcag/gwab049
Article ID: gwab049
Article version: 1
Subjects: Author Index, AcademicSubjects/MED00260

Author Index

Publication date: 2022 Mar
Electronic publication date: 2022 Feb 21
Volume: 5
Issue: Suppl 1
Issue title: Abstracts Accepted to 2022 CDDW™
First page: 157
Last page: 181
Copyright: ڣ The Author(s) 2022. Published by Oxford University Press on behalf of the Canadian Association of Gastroenterology.
Copyright year: 2022
License: This is an Open Access article distributed under the terms of the Creative Commons Attribution License (https://creativecommons.org/licenses/by/4.0/), which permits unrestricted reuse, distribution, and reproduction in any medium, provided the original work is properly cited.
License URL: https://creativecommons.org/licenses/by/4.0/

Page count: 25

==============================

A

Abdelsalam, O A138
Abduallah, Y A150
Abdullah, N A230, A52
Abraldes, J A223
Abreu, M A238
Achiron, R A221
Adekunle, F A209
Adeyi, O A220
Afif, W A143, A150, A180
Afzaal, T A227
Afzal, N A128, A129
Agosti, F A52
Aithal, G A203
Akhtar, D A101, A136, A155, A179, A258, A59
Akhtar, N H. A136, A179, A258, A59
Akman, O A109
Alajmi, A A61
Alazemi, K A103
Albader, F A180
Albalawi, Z A74
Albino, L A146
Al Fedaghi, A A61
Ali, A A190
aljabri, R s. A114
Allain, T A15, A56
Allaire, J M. A1, A2, A40, A9
Allison, M A203
Almadi, M A27
Al Mazrou'i, A A213
Almeida, P A185
Almeshal, F A259
Almousawi, F A125
Almudaires, A A106
Almutairi, K A18
Alobaid, S A71
Alqahtani, S A106
Alsager, M A218
Alshihry, I A. A126
Altamimi, E M. A260
Altier, C A230, A36, A52
Al-Zubide, S A133
Ambrosio, L A175
Amin, S A32
Anderson, J A107
Andrews, C N. A134

Anglin, R A229
Anstee, Q M. A219
Anvari, S A83
ApSimon, M A251
Arab, J A203
Arai, T A11
Ariaratnam, J A25
Arif, A A. A105, A123
Arjomand Fard, N A237
Armstrong, D A112, A192, A194, A196, A250, A251, A255
Armstrong, H A229, A47, A50
Arsenault, A M. A183
Asfaha, S A166, A35
Aspinall, A A202
Atkinson, K A179
Aumais, g A157
Aumais, G A236
Avinashi, V A130
Avni-Biron, I A236

B
Bachand, M A197
Baert, F A37
Baggio, C H. A64
Bah, B A81
BAI, X A6
Bailey, R A217, A223
Baillargeon, D A254
Baird, R A222
Bak, A W. A95
Bansal, R A25, A89, A92, A99
Barker, C A179, A189
Barker, M A91
Barkun, A N. A107, A27, A33, A94
Barkun, C A94
Barnett, J A. A49
Baugmart, D C. A146, A160, A57, A93
Beaton, M D. A142
Beaulieu, J A19, A58
Beaupre, E A198
Begum, J A159, A162
Belletrutti, P J. A118, A124
Belmesk, L A253
Bemeur, C A244
Benchaya, J A33
Benchimol, E I. A159, A162, A23

Benmassaoud, A A110
Benoit, N A141
Berard, R A190
Bercik, P A13, A14, A144, A196, A229, A248, A34, A46, A51, A6
Berenguer, M A209
Berg, S A37
Bernstein, C N. A154, A157, A159, A162, A164, A23, A236, A238
Bertelle, V A19
Bessissow, A A204
Bessissow, T A143, A150, A180
Beyak, M J. A133, A172, A80
Bhandal, S A69
Bhanji, R A224, A226
Bhoey, P A102
Biertho, L A254
Bihari, A A65
Bilal, M A32
Bindra, G A97
Bitton, A A143, A150, A180
Blakely, P A127
Boisvert, F A3, A7
Bollipo, S A32
Bording-Jorgensen, M A237, A47, A50
Borgaonkar, M A170
Borman, M A202
Borojevic, R A248, A51
Borowski, K A156, A161, A235
Bortolin, K A128
Bortolin, K A. A129
Boudreau, F A21
bouin, m A107, A132, A244
Bourke, M A111
Bouthot, J A253
Boutros, M A141
Bouvet, L A254
Bowdish, D A14
Brackenridge, E A66
brahmania, m A203, A217, A218, A224, A225
Brar, G S. A152
Bray, C A88
Bressler, B A179
Brill, H A66
Brumer, H A38
Brundler, M A69
Budhram, D A79
Bunnett, N W. A53

Burak, K W. A202
Burak, K A223
Buret, A A15, A56
Burgess, N A111
Butcher, J A233, A234
Buttar, J A101, A179
Byrne, M A113

C

Cai, B A163
Callejas Pina, B E. A60
Calman T'ien, L A75
Cambron-Asselin, C A186, A187, A188
CAMINERO FERNANDEZ, A A46, A48
Capozza, T A219
Carbone, M A209
Carbonneau, M A88
Carman, N A70
Carrión-Jaramillo, E A130
Carroll, M W. A47
Case, S A193
Cassidy, C A26
Causada-Calo, N A248
Cave, K A116
Cepo, J A177
Cestari, I A4
Chadee, K A198, A43, A63
Chaitanya, V A218
Chakroborty, A A250
Chan, D A74
Chan, M A148
Chande, N A142, A166
Charabaty, A A32
Chau, S A184
Chauhan, U A66
Chekkal, A A22
Chen, T A110
Chen, Y A40
Chen, Y A110, A204, A33
Chhibba, T A24
Chiew, B A. A73
Chin Koon Siw, K A148, A151, A153
Chirdo, F G. A46, A5
Chiu, E A54
Choi, M A214
Chow, J A224, A226

Cino, M A236
Cipriani, T A64
Clarke, A A191
Cloutier, G A58
Coburn, N A257
Coffin, C S. A202, A217
Cohen, A A141
Cohn, I A129
Collins, A W. A255
Collins, S M. A13, A14, A229, A51, A6
Colombel, J A167, A37
Congly, S E. A202, A217
Connors, J M. A183
Constante, M A10, A144, A200, A46
Coombes, B K. A200
Cooper, C A217
Cooper, J A211
Cote, M A254
Courbette, O A186, A187, A188
Couture, C A254
Coward, S A158, A211
Croitoru, K A157, A238
Croitoru, K A236
Cronin, O A111
Crowley, E A190, A42
Crowley, S M. A2, A40, A9
Cumenal, M A230, A52
Currie, B A26, A96

D

D'Haens, G A145, A37
D'Silva, A A247
Dadgar, K A152
Dal Soglio, D A186, A187, A188
Danese, S A145
Dang, F A169
Dang, J A127
Daniels, V J. A78, A87
Daperno, M A167
Da Silva, S A128, A129
Davila-Cervantes, A A93
deBruyn, J A23
de Buck van Overstraeten, A A153
Decanini-Trevino, J A93
Defaye, M A230, A36, A52
Degro, C E. A53

Dehbidi Assadzadeh, L A22, A253
Delawarde-Saïas, C A244
de-Madaria, E A32
De Palma, G A13, A14, A229, A51, A6
Desai, T A70
Deschenes, M A110
Desgroseilliers, C A141
Deshpande, A A174
Desilets, E A132
Deslandres, C A186, A187, A188, A22, A236, A253, A81
Devic, S A141
Diarra, A A182
Didar, T A5
Diec, H A141
Dieleman, L A. A146, A160, A175, A47, A57
Dijk, S I. A176
Dinn, S E. A69
Dionne, J A227
Dirks, M A186, A187, A188, A22
Djani, L A186, A187, A188
Djerboua, M A215
Djinbachian, R A107, A30
Djoukam Mbuko, B A22
Dobranowski, P A234
Donaldson, K A105, A123
Donaldson, M A. A101, A136, A258
Donnellan, F A113, A136, A256, A59, A86
Dotan, I A236
Doucette, k A217
Douchin, J A3
Dowhaniuk, J A194
Dowhaniuk, J K. A66
Droubi, B A260
Drouin, É A186, A187, A188
Drügg Hahn, G A143, A180
Du, L A146
Duan, W A37
Dube, C A108
Dubey, A A17
Dubinsky, M A37
Dubois, S A213
Duerksen, D A193, A252
Dufour, A A230, A3
Dufour, J A219
Dulai, P S. A167
Dunsmore, G A175

Dziegielewski, C A168

E
Ebadi, M A224, A226
Edwards, M A102
Eickhoff, A A30
Eindor, A A28
Eissa, N A182
Elahi, S A175
Elkhashab, M A217
El-Matary, W A23, A236
Elsolh, K A104
Eltorki, M A241
Enderle, M D. A30
Engel, J A153
Enger, S A121, A141
Enns, R A. A75
Espin Garcia, O A238
Espin-Garcia, O A157, A236

F
Fadela Zekhnine, S A186, A187, A188
Faisal, M A194, A255
Fardy, J A170
Faria, J A141
Farina, D A122
Fathi, A A8
Feagan, B G. A236, A37
Feagan, B A42
Fecso, A A115, A92
Feizi Farivar, J A214
Fekete, E A15, A56
Ferrante, M A37
Ferretti, E A19
Ferri, L A33
Figeys, D A234
Finney, C A232
Flannigan, K L. A12, A20
Flemming, J A215, A29
Focht, G A176
Fontes, M E. A46
Forbes, N A124, A205
Francheville, J A82
Fredriksen, M A249
Friedman, G A141
Frolkis, A A202, A24, A97

Fujiyoshi, M A. A25
Fung, M A148
Fung, S A217

G
Gagné, D A19
Galiatsatos, P A141
Galipeau, H J. A10, A144, A200, A46, A5
Gallant, A A22
Gallinger, Z A117, A169
Galorport, C A149, A75
Gan, S A113, A258
Gandhi, R A218
Garant, A A121, A141
Gartke, T A50
Geary, T G. A11
Geng, S A253
Genis, S A89
Ghia, J A182
Gholami, R A25
Ghosh, S A54
Ghosh, S M. A78, A87
Ghosh, S A138
Gibson, D L. A49
Gidrewicz, D A193, A67
Gill, S K. A49
Gimpaya, N A104, A140, A25, A32, A89, A92, A99
Girard, C A186, A187, A188
Giroux, V A16, A17, A21, A3
Godbout, J A10
Goethel, A A157, A236, A238
Goldshtein, D A29
Golovics, P A143, A150, A180
Gomes, T A159, A162
Gong, R A81
Gonneaud, A A16, A17, A3
Good, H J. A35
Goodman, K A65
Gorenko-Lévêque, S A22
Gorman, H A198, A43
Gould, M A245
Govardhanam, V A177, A178, A76
Gowing, A A234
Gozdzik, M A151
Gramlich, L A249
Grant, C A66

Gravel, J A22
Greaves, G E. A95
Greenaway, M A100
Greenblatt, M A254
Gregor, J C. A109, A142, A166
Greiner, R A176
Griffiths, A A157, A176, A23, A236, A238, A42
Groleau, V A186, A187, A188, A22
Grover, S A104, A120, A140, A25, A32, A89, A92, A99
Gruchy, S E. A122
Gryspan, D A19
Grzywacz, K A186, A187, A188
Guizzetti, L A71
Gulamhusein, A A210
Guo, C A184
Guo, H A118
Guo, J A116
Gupta, M A134
Gupta, S A115, A140, A168
Gupta, S A111
Gupta, V A192
Guttman, O A189
Guttmen, D S. A236, A238
Guzman Rodriguez, M A242

H
Haas, D A48
Habashi, P A169
haberman, y A221
Hagström, H A203
Halac, U A186, A187, A188
Halder, S L. A66
Halloran, B A116, A146, A160, A31, A57, A93
Halloran, P A160, A57
Hamed, S A60
Hamilton, M A21
Han, X A2, A40, A9
Hann, A A144, A200, A5
Hanna, Q A172
Hanna, Y A177
hansen, b A210
Harding, K G. A95
Harris, A A256
Hart, A A153
Hasan, S A119
Hashim, A A32

Haskey, N A49
Hassan, A A230, A52
Healey, G R. A228
Heatherington, J A97
Hébuterne, X A145
Heisler, C A26, A77, A96
Heitman, S A124, A205
Hernandez-Rocha, C A156, A161, A235, A238
Higgins, P A145
Hindi, Z A203
Hirota, S A. A12, A20
Hirschfield, G A209, A210
Hisamatsu, T A37
Hong, Z A120
Hookey, L A102, A259, A79
Hosseinidoust, Z A200
Hotte, N A175
Hould, F A254
Hsu, A A257
Huang, B A37
Huang, R A224
Huang, V A174, A175, A177, A178, A76
Huard, G A224
Huard, T A26
Hudson, A A185
Hudson, D A103, A225
Hulst, J A128, A129
Hussain, M A103
Huynh, H Q. A157, A185, A23, A236, A238, A47
Hyams, J A236
Hyde, A A171, A208, A78, A88
Hynes, A A6

I
Iablokov, V A166
Iannuzzi, J A74
Iftinca, M A230, A52
Ilnyckyj, A A119, A173
Ingram, R A54
INVESTIGATORS, P A94
Ismail, M A210
Issenman, R A66
Ito, S A129

J
Jackson, K A144, A200

Jacobson, K A1, A157, A228, A23, A236, A238, A28, A45
Jain, A A91
Jairath, V A166, A42, A71
Jalali, S A207
Jaleel, S A41
James, P D. A205
James, P A257
JAMES, L J. A212
Janisse, N A251
Jantchou, P A22, A253, A81
Jantchou, P A186, A187, A188
Jaramillo Polanco, J A240
Jardim, A A11, A4
Jardine, S A184
Jean, D A16, A21
Jelinski, S A100
Jeyalingam, T A124
Jeyaparan, J A217
Jhangra, R A83
Jiang, S X. A137, A214, A256
Jijon, H A97
Jiménez-Vargas, N N. A242, A243, A53
Jin, J A249
Jivraj, A A192
Jogendran, R A76
Johar, J A122
Johnson, E A208, A78, A87, A88
Jones, D A209
Jones, G A251
Jones, J A139, A26, A77, A84, A96
Jovel, J A47
Jowhari, F A95
Jubran, B A210
Jugnundan, S A115, A120
Jung, H A38, A39
Jurencak, R A70
Jurkovic, C A7
Juurlink, D A159, A162

K

Kafyeke, R A81
Kaplan, G G. A158, A159, A162, A211, A97
Karachi, T A251
Karachiwala, H A138
Karimi, P A246, A68
Karmali, S A127

Karvellas, C J. A227
Kassif, E A221
Kataria, J A131
Kaviani, R A90
Keane, K P. A18
Kelley, M A79
Khalfaoui, T A58
Khalidi, N A196
Khan, R A104, A140, A25, A257, A32, A89, A92, A99
Khan, U A205
Khanna, R A103, A142, A166, A225
Khaouli, M A193
Kherad, O A27
Khosrow-Khavar, F A141
Kilfoil, E A139
Kim, A A43
Kim, B A125
Kim, R B. A142
Klarenbach, S A158
Klemm, N K. A98
Kligys, K A37
Ko, H A214
Kobor, M A8
Kohansal, A R. A122, A205
Koletzko, S A176
Komeylian, H A96
Kosick, H M. A220
Koto, P A82
Krahn, T A31, A93
Kraimi, N A14
Kral, J A32
Kremer, A E. A209
Kroeker, K A146, A158, A160, A171, A57, A65, A93
Kuenzig, E A159, A162
Kulyk, A A165
Kundapur, P A173
kwan, p A114

L

Laforce, S A244
Laforest-Lapointe, I A231
Lai, J A223
Lakatos, P L. A143, A150, A180
Lam, E A105, A123
Lamarche, B A244
Lan Thai, H A141

Lapointe, A A22
Larsen, F A35
Lavalle, C A112
Lavoie, P A1, A45
Lawrence, S A45
Lee, E A111
Lee, J G. A85
Lee, J A20
Lee, S A202
Lee, S A106
Lee, S A157, A161, A235, A236, A238
Leibovitzh, H A157, A161, A235, A238
Leon-Coria, A A232
Leong, R A241, A246, A68
Leroux, L A11
Leroy, V A209
Letellier, A A72
Leung, V T. A137
Li, D Y. A134
Li, J A233
Li, J A89, A92
Li, S A181
Liang, Q A9
Liao, X A37
Libertucci, J A248, A46
Likhitsup, A A203
Lim, A A171
Lim, D R. A212
Lin, D A8
Lin, Y A186, A187, A188
Lindsay, J A145
Linton, J A48
Lisondra, J A25
Lithgow, K A74
Little, D H. A210
Liu, J A145
Lochnan, H A. A191
loftus, E A145
Lomax, A E. A239, A240, A242, A243, A53
Loomba, R A219
Loonen, L A248
Lopes, F A11, A4, A41, A44
Lopez Lopez, C A240
Lorentzian, A A2
Low, D J. A120
Lu, C A146

Lu, J A13, A14, A229
Lucena Périco, L A62
Lui, R N. A32
Luyun, P A214
Luzentales-Simpson, M A38

M
Ma, C A199
Ma, C A146, A158, A211, A24, A42
Ma, M A217, A226
Ma, W A38
MacIntosh, D A139
MacIsaac, J A214
Mack, D R. A157, A23, A233, A234, A236, A238
MacNaughton, W A62, A64
Madagh, S A253
Madill-Thomsen, K A160, A57
Madrigal, A G. A4
Madsen, K A157, A171, A175, A238
Mahmud, F A191
Malacco, N O. A4
Malik, G A206
Mallick, R A153
Maniere, T A132
Manko, A A217
Manoogian, J A233
Marchand, V A186, A187, A188
Marcon, P A128, A129, A191, A245
Markovinovic, A A211
Marshall, D A247
Marshall, J A157, A167, A236, A238, A66
Martel, M A27, A33, A94
Martin, A A121
Martin, R J. A11
Martinez-Lozano, H A161, A235
Martins, K A158
Mason, A A208
Mason, J A159, A162
Masson, S A203
Maxwell, C A2
Maxwell, C A174, A177
Mayne, J A234
McCall, M A216
McCoy, K A230, A54
McCune, A A203
McCurdy, J A148, A151, A152, A153, A168

Mcdonald, C A106, A71
McDonald, C A191
McGrath, J S. A170
McIntosh, K A142
Mckay, D M. A181, A232, A55, A56, A60
McKay, H A42
McLean, R A127
McLeod, M A208
Meddings, J A157, A238
Mehta, K A194
Meier, T A88
Meleady, L A28
Meng, Z A205
Menzies, S A38, A39
Metellus, D A107
Michael, F A213
Michael, H A213
Miles, M A134
Miller, C S. A141, A33
Miller, C A121
minuk, g A217
Miranda, P M. A34
Mistry, J A193
Moayyedi, P A157, A172, A236, A238
Mohan, V A51
Monod, A A108
Montano-Loza, A J. A224, A226, A93
Moreau, F A198, A43, A63
Morrin, L A100
Morsa, M A81
Mosko, J A115, A125, A32
Moss, K A194
Motomura, D A113
Moysey, B A100
Mrkobrada, M A250
Mslati, M A199
Muise, A A184, A42
Mukaneza, Y A244
Mukherjee, A A120
Murray, T A137
Murthy, S A168
Muthiah, K A165

N
Nadeau, M A254
Nair, A A70

Nakase, H A145
Nardelli, A A248, A51
Narous, M A164
Narula, N A167, A66
Nascimento, A A64
Nasser, Y A134, A247, A97
Natt, N A213
NavaneethaKrishnan, S A55
Navas-López, V A176
Nawawi, A A204
Nayeri, S A156, A161, A235
Neary, E A104, A242, A243, A79
Neil, E A26
Neimark, E A37
Nemecek, N A100
Nettleton, J E. A183
Neumark, S A83
Neustaeter, A A157
Nguyen, D M. A177
Nguyen, G C. A169
Nguyen, H H. A202
Nguyen, T A42
Nguyen, V A141
Nguyen, V V. A223
Nguyen, V C. A95
Ngwanou, D A22
Nimmo, M A59
Nogueira de Almeida, L A3
Novak, K L. A211, A97
Nugent, Z A159, A162, A164

O
O'Connor, K A174
Ohland, C L. A230
Oilund, C A100
Olaiya, O A61
Oliveira Ponce, J P. A44
Olivera Sendra, P A. A161, A235
Omar, M A224
Oshiomogho, J A97
Otley, A A23, A236
Otley, M O. A183
Ou, G A137, A85
Owen, D A256, A258
O’Sullivan, T A111

P

Pace, K A191
Pai, N A66
Painchaud, M A147
Panaccione, R A118, A145, A157, A158, A211, A236, A37, A54, A73, A97
Panes, J A145, A37
Pang, Y A37
Pangan, A A145
Paradis, A A197
Parfitt, J A218
Parker, B A95
Parker, R A203
Parkins, V A74
Parvizian, M K. A102
Pasieka, J A74
Pasumarthi, K A83
Patel, A A250
Patel, K A220
Patel, S A122
Paterson, W G. A133, A80
Patey, N A186, A187, A188
Pattni, C A115, A25, A89
Pawlak, K M. A32
Peerani, F A146, A160, A171, A57
Pekar Zlotin, M A221
Peltekian, K A82
PELTEKIAN, K M. A212
Peña-Sánchez, J A159, A162
Penner, R M. A147
Peretz, D A224
Perini, R A95
Perreault, N A7
Perren, J A95
Perry, T A237
Petrova, A A47
Peyrin-Biroulet, L A37
Phalen-Kelly, K A26, A96
Piché, N A22
Pierre, N A22
Pinto-Sanchez, M I. A192
Pinto-Sanchez, M I. A193, A194, A248, A251, A255, A34, A46, A51
Piper, H A1
Piskin, D A190
Pivorunas, V A37
Pohl, H A107
Pohoreski, K A67

Poloni, C A8
Pomerleau, V A7
Pon, S A160, A57
Ponich, T A142
Poon, B A1
Pray, C A167
Prevot, T A14
Prowse, K A66
Pujo, J A13
Puka, K A218
Punthakee, Z A191

Q
Qaddouri, M A186, A187, A188
Qian, H A105, A123
Qin, H A234
Qumosani, K A218, A225

R
Rabbia, V A229
Radoja, C A66
Rahman, A A250
Rahman, S H. A217
Rahmani, S A5
Rai, M A259
Rajagopalan, V A247
Rajeev, S A181, A232
Rajoriya, N A203
Raman, M A247, A54, A73
Ramay, H A54
Ramirez-GarciaLuna, J A246
Ramji, A A214, A217, A219
Ramkissoon, A A25
Randall, D A134
Ratcliffe, E A241, A246, A66, A68
Ratziu, V A219
Raygoza Garay, J A236, A238
Reed, D E. A133, A135, A239, A240, A242, A243, A53, A80
Rees, W D. A8
Reicherz, F A45
Reid, J A149
Reimer, R A54
Reinisch, W A167
Restellini, S A27
Reyes-Nicolas, V A7
Ribeiro, T A10

Ricci, M A135
Richardson, W A95
Richer, L A158
Rinella, M A219
Rivera, D A131
Robar, J A26
Rodrigues, D A102, A131, A135, A80
Rogalsky, A A245
Rohatinsky, N A26
Roizes, S A18
Rolland, M A16
Rolland, S A72
Rondeau, L A48
Rosenfeld, G A149
Rosentreter, R A146
Rossi, L M. A46
Rostom, A A108
Rotermund, C A30
Rueda, G H. A248, A34, A46
Ruether, D A74
Russell, L A194, A251, A255

S
Saad Mohammed, A A259, A29
Sabri, E A148
Sadler, M D. A202
Safari, K A39
Saibil, F A191
Salh, B A101, A155, A201, A98
Salim, M A205, A257
Saloojee, N A168
Sanchez gonzalez, Y A145
Sanchez-Luna, S A32
Sandborn, W A145, A37
Sandhu, D S. A32
Sandre, A A83
Santiago, A A144
Sanyal, A J. A219
Saqib, Z A6
Sarbar, S A29
Sarker, A A151
Sassine, S A186, A187, A188
Savoie-Robichaud, M A186, A187, A188
Sawhnry, S A74
Scaffidi, M A104, A140, A25, A89, A92, A99
scharr, Y A217

Schick, A A228
Schmidt, M A190
Schneider, R A42
Schreiber, R A222
Schreiber, S A37
Scodellaro, S A129
Sebastiani, G A110, A220
Seiler, C A34
Seleq, S A140
Seleq, S A104, A25, A32
Seow, C A158, A97
Serra, A A12
Serrano, P E. A251
Sethuraman, A A189
Sey, M A106, A142, A71
Shaheen, A M. A202, A211
Shahidi, N C. A105, A111, A123, A126, A90
Shajari, E A19
Shang, J A64
Sharafianardakani, Z A1
Sharkey, K A. A36
Sharma, R A66
Sharma, S A77, A84
Shelton, J A216
Shengir, M A220
Shepherd, T A26
Shergill, A A92
Sherlock, M A66
Shi, N A203
Shin, A E. A35
Shire, Z A45
Siau, K A32
Sibille, E A14
Siciliani, E A. A11, A4
Sidhu, M A111
Siebring, V A106
Siffledeen, J A146, A171
Silverberg, A A42
Silverberg, M S. A156, A161, A235, A236
Sinal, C A183
Singh, H A109, A164
Singh, K S. A121
Singh, N A226
Singh, S A147
Singh, S A223
Singleton, R A234

Skaro, A A218
Skinn, A A189
Sljivic, I A86
Sloboda, D A10
Sly, L M. A38, A39
Smith, B C. A201
Smith, M K. A224
Smith, M I. A236
Smyth, M A222
Snider, J A100
Sokol, H A248
Song, A A37
Sosnowski, O A56
Southward, C M. A46
Spankie, C A100
Spence, J C. A208
Spiers, J A88
Squirell, E A170
Stach, J A118
Stadnyk, A A183
Steg Saban, O A221
Stein, C A53
Stein, M A210
Steinhart, H A157, A174, A236
Stempak, J A156, A161
Stephanie, H A250
Stephens, M A18
Stevens, M A252
Stevenson, M A11, A4
Stewart, M J. A139, A77
Stewart, M A26, A84, A96
Stinton, L A202
Stintzi, A A233, A234
Stone, J A154
St-Pierre, J A97
Strickland, M A216
Su, S A154
Suarez Suarez, R G. A176
Suliman, M I. A31
Sullivan, R A139
Sulymka, B A252
Sun, W A127
Sunil, M A246, A68
Surette, M A229, A46, A6
Svendsen, K A36
Swain, M A202, A211

Sy, R A168
Szamosi, J C. A46
Szczepanski, H E. A20
Szilagyi, A A195

T
Taghiakbari, M A107, A30
Tai, T A95
Talarico, R A257
Tam, E A214
Tam, L A155, A59
Tam, M A11
Tan, J A196
Tandon, P A174, A175, A177, A178, A76
Tandon, P A171, A223, A73, A78, A87, A88
Taneja, S A223
Tang, E A218
Tanuseputro, P A257
Tao, J J. A177, A178
Targownik, L E. A158, A159, A162, A26
Tate, D A111
Taylor, L A54, A73
Tchernof, A A254
Ted, S A8
Telford, J J. A75, A91
Telkar, N A8
Teriaky, a A218, A225
Teshima, C W. A115
Tham, D A25
Thibault, M A19
Thibodeau-Antonacci, A A121
Tilbe, K A112
Timpano, J A157
Tomaszewski, M A126, A256, A90
Torabi, N A92
Towfighi, S A256
Trasolini, R A86, A98
Tremblay, É A19
Tremblay, M A244
Tripp, D A. A172
Trylesinki, A A219
Tsai, K A2
Tsang, L A249
Tsang, Q K. A239, A53
Tshikudi, D M. A182
Tsoi, K A207

Turner, D A176, A236, A238, A42
Turner, J A193
Turpin, W A156, A157, A236, A238

U
Underwood, F A100
Unninayar, D A151
Urban, J F. A11

V
Valcheva, R A47
Valenti, D A204
Vali, R A245
Vallance, B A1, A199, A2, A228, A40, A9
Vallance, J A247
Vallis, M A26
Vander Well, S A208
van Hattem, W A111
Vanner, S A239, A240, A242, A243, A53, A80
Vantomme, E A252
Vargas, J I. A32
Vasilevsky, C A141
Veniamin, S A237
Venner, J A160, A57
Verdu, E A10, A14, A144, A192, A194, A200, A229, A248, A46, A5
Verdugo Meza, A A49
Verma, Y A99
Vermeire, S A145
Verstegen, R A129
Vigod, S A159, A162
Visva, S A153
von der Weid, P A18
von Renteln, D A107, A30
Vosko, S A111
Vu, D C. A152
Vukovic, S A117
Vuong, T A141
Vuong, T A121
Vurzinger, M A130

W
Walker, K A234
Wallace, K A37
Walters, T A23
Wang, A A181, A232, A55, A60
Wang, C A38

Wang, C A138, A93
Wang, D A207
Wang, G A175
Wang, P A215
Wang, S A223
Wang, S A63
Wang, X A48
Warner, N A184
Wasilenko, S A31, A93
Watt, M A171, A208
Webb, J A237
Weber, A A81
Weinreich, M A145
Weishaupt, L L. A121
Weiss, B A221
Weissbach, T A221
Weiten, D A193
Wells, J A248
Wetwittayakhlang, P A143, A180
Whitfield, A A111
Whitlock, R A223
Wild, G A143, A150, A180
Wild, T C. A78
Williams, C A139
Williams, G A122
Williams, S A111
Wilson, A A163
Wilson, A A142
Wilson, H A. A127
Wine, E A176, A237, A47, A50, A65
Wishart, D S. A176
Witt, J A23
wong, a A217
wong, S A98
Wong, C A127
Wong, E C. A167
Wong, K A146, A160, A206, A57
Wong, M A8
Wong, P A110, A217, A224
Wong, S A29
Woo, M A134
Wozney, L A26
Wright, G M. A208
Wrobel, I A69
Wu, R Y. A175
Wulczynski, M A10

Wyse, J A33

X
Xenodemetropoulos, T A83
Xiao, Y A205
Xie, W A145
Xu, C A223
Xu, W A157, A236, A238
Xue, M A157, A238
Xue, X A195

Y
Yaghoobi, M A112
Yan, B A106, A71
Yang, D Y. A206, A93
Yang, H A136
Yang, H A40
Yanofsky, R A150
Ye, J A49
Yee, B A95
Yeo, C A74
Yoshida, E M. A212, A224
Young, D A230
Younossi, Z A219
Yu, H A199, A9
Yu, Y A239, A242, A53

Z
Zachos, M A66
Zachos, N A8
Zamponi, G W. A52
Zeidan, E A132
Zepeda-Gomez, S A116, A31, A93
Zhang, L A35
Zhang, M A102
Zhang, Z A176, A47
Zhang, Z A52
Zhao, X A110
Zhao, Y A22
Zhouwen, W A145
Zhu, J A212
Zhu, K A98
Zogopoulos, G A204
Zoughlami, A A204
Zuniga-Villanueva, G A246, A68
